# Validation of Inference Procedures for Gene Regulatory Networks

**DOI:** 10.2174/138920207783406505

**Published:** 2007-09

**Authors:** Edward R Dougherty

**Affiliations:** Department of Electrical and Computer Engineering, Texas A&M University; Computational Biology Division, Translational Genomics Research Institute; Department of Pathology, University of Texas M.D. Anderson Cancer Center, USA

**Keywords:** Epistemology, gene network, inference, validation.

## Abstract

The availability of high-throughput genomic data has motivated the development of numerous algorithms to infer gene regulatory networks. The validity of an inference procedure must be evaluated relative to its ability to infer a model network close to the ground-truth network from which the data have been generated. The input to an inference algorithm is a sample set of data and its output is a network. Since input, output, and algorithm are mathematical structures, the validity of an inference algorithm is a mathematical issue. This paper formulates validation in terms of a semi-metric distance between two networks, or the distance between two structures of the same kind deduced from the networks, such as their steady-state distributions or regulatory graphs. The paper sets up the validation framework, provides examples of distance functions, and applies them to some discrete Markov network models. It also considers approximate validation methods based on data for which the generating network is not known, the kind of situation one faces when using real data.

## 1. INTRODUCTION

The construction of gene regulatory networks is among the most important problems in systems biology [1-2]. Network models provide quantitative knowledge concerning gene regulation and, from a translational perspective, they provide a basis for mathematical analyses leading to systems based therapeutic strategies [3]. Network models run the gamut from coarse-grained discrete networks to the detailed description of stochastic differential equations. The availability of high-throughput genomic data has motivated the development of numerous inference algorithms. The performance, or validity, of these algorithms must be quantified. An inference algorithm takes a sample set of data as input and outputs a model network. Its validity must be evaluated relative to its ability to infer a model network close to the ground-truth network from which the data have been generated. Given a hypothetical model, generate data from the model, apply the inference procedure to construct an inferred model, and compare the hypothetical and inferred models *via *some objective function.

This paper mathematically formulates validation in terms of the distance between two networks, or the distance between two structures of the same kind deduced from the networks, such as their steady-state distributions. As a function from a sample set to a class of network models, an inference procedure is a mathematical operator and its performance must be evaluated within a mathematical framework, in this case, distance functions. The paper sets up the validation framework in general terms, provides examples of distance functions, and applies them to some basic network models. It also considers approximate validation methods based on data for which the generating network is not known, the situation one faces when using real data.

It is hoped that this paper will help to motivate the study of network validation procedures. While we believe it describes the general setting and the basic requirements for validation, as will be pointed out, to date, there has been very little study devoted to network validation. There are many subtle statistical issues. If we are to be able to judge the worth of proposed algorithms, then these issues need to be addressed within a formal mathematical framework.

## 2. BACKGROUND: NETWORK MODELS

Although our aim is to consider network inference from a fairly general perspective, to give concrete examples we require some specific models. Thus, we assume the underlying network structure is composed of a finite node (gene) set, *V* = {*X*_1_, *X*_2_,…, *X_n_*}, with each node taking discrete values in [0, *d* – 1]. The corresponding state space possesses *N* = *d^n^* states, which we denote by **x**_1_, **x**_2_,…, **x**_*N*_. We express the state **x**_*j*_ in vector form by **x**_*j*_ = (*x_j_*_1_, *x_j_*_2_,…, *x_jn_*). For notational convenience we write vectors in row form but treat them as columns when multiplied by a matrix. The corresponding dynamical system is based on discrete time, *t* = 0, 1, 2,…, with the state-vector transition **X**(*t*) → **X**(*t* + 1) at each time instant. The state **X **= (*X*_1_, *X*_2_,…, *X_n_*) is often referred to as a gene activity profile (GAP).

### Markov Chains

We assume that the process **X**(*t*) is a *Markov chain*, meaning that the probability of **X**(*t*) conditioned on **X** at *t*_1_ < *t*_2_ < … < *t_s_* < *t* is equal to the probability of **X**(*t*) conditioned on **X**(*t_s_*). We also assume the process is *homogeneous*, meaning that the transition probabilities depend only on the time difference, that is, for any *t* and *u*, the *u*-step transition probability,

(1) $$p_{\mathrm{jk}}\left(u\right)=P\left(X\left(t+u\right)=x_{k}|X\left(t\right)=x_{j}\right)$$

depends only on *u*. We are not asserting that the Markov property and homogeneity are necessary assumptions for gene regulatory networks. We make these assumptions to facilitate mathematically tractable modeling for the current study. Under these assumptions, we need only consider the one-step transition probability matrix,


                (2)
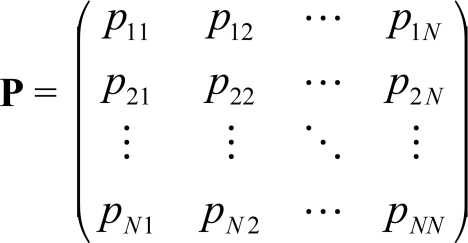

				

where the one-step transition probability, *p_jk_*, is given by *p_jk_* = *p_jk_*(1). We refer to **P** simply as the *transition probability matrix*. For *t* = 0, 1, 2,..., the state probability structure of the network is given by the *t*-state probability vector

(3) $$\text{p}\left(t\right)=\left(p_{1}\left(t\right),p_{2}\left(t\right),...,p_{N}\left(t\right)\right)$$

where *p_j_*(*t*) = *P*(**X**(*t*) = **x**_j_). **p**(0) is the initial-state probability vector.

Besides the state one-step probabilities, we can consider the gene one-step probabilities,

(4) $$p_{i}\left(j,r\right)=P\left(X_{i}\left(t+1\right)=r|X\left(t\right)={\text{x}}_{j}\right)$$

If, given the GAP at *t*, the conditional probabilities of the genes are independent, then

(5) $$p_{\mathrm{jk}}=p_{1}\left(x_{\mathrm{j1}},{\text{x}}_{k}\right)p_{2}\left(x_{\mathrm{j2}},{\text{x}}_{k}\right)...p_{n}\left(x_{\mathrm{jn}},x_{k}\right)$$

Suppose that gene *X_i_* at time *t* + 1 depends only on values of genes (predictors) in a regulatory set, $R_{i}\subset V$ at time *t*, the dependency being independent of *t*. Then the gene one-step probabilities are given by

(6) $$p_{i}\left(j,r\right)=P\left(X_{i}\left(t+1\right)=r|X_{l}\left(t\right)=x_{\mathrm{jl}}\text{ for }X_{l}\in R_{i}\right)$$

In this form, we see that the Markov dependencies are restricted to regulatory genes.

The network has a *regulatory graph* consisting of the *n* genes and a directed edge from gene *x*_i_ to gene *x*_j_ if $x_{i}\in R_{j}$. There is also a *state-transition graph* whose nodes are the *N* state vectors. There is a directed edge from state **x**_*j*_ to state **x**_*k*_ if and only if **x**_*j*_ = **X**(*t*) implies **x**_*k*_ = **X**(*t* + 1).

A homogeneous, discrete-time Markov chain with state space {**x**_1_, **x**_2_,…, **x**_*N*_} possesses a steady-state distribution (π_1_, π_2_,…, π_*N*_) if, for all pairs of states **x**_*k*_ and **x**_*j*_, *p_jk_*(*u*) → π_*k*_ as *u* → ∞. If there exists a steady-state distribution, then, regardless of the state **x**_*k*_, the probability of the Markov chain being in state **x**_*k*_ in the long run is π_*k*_. In particular, for any initial distribution **p**(0), *p_k_*(*t*) → π_*k*_ as *t* → ∞. Not all Markov chains possess steady-state distributions.

### Rule-Based Networks

A basic type of regulatory model occurs when the transition **X**(*t*) → **X**(*t* + 1) is governed by a rule-based structure, meaning there exists a state function **f** = (*f*_1_, *f*_2_,…, *f_n_*) such that *X_i_*(*t* + 1) = *f_i_*(*R_i_*(*t*)). A classical example of a rule-based network is a Boolean network (BN), where the values are binary, 0 or 1, and the function *f_i_* can be defined *via *a logic expression or a truth table consisting of 2^*n*^ rows, with each row assigning a 0 or 1 as the value for the GAP defined by the row [4-5]. As defined, the BN is deterministic and the entries in its transition probability matrix are either 0 or 1. The *connectivity* of the BN is the maximum number of predictors for a gene. If each has the same number of predictors, then we say that the network has uniform connectivity.

The model becomes stochastic if the BN is subject to perturbation, meaning that at any time point, instead of necessarily being governed by the state function **f** = (*f*_1_, *f*_2_,…, *f_n_*), there is a positive probability *p* < 1 that the GAP may randomly switch to another GAP. There are more refined ways of characterizing perturbations, such as defining perturbations at the gene level rather than the state level, but state-level perturbation is easy to describe and is sufficient for our purposes here. For a BN with perturbation, the corresponding Markov chain possesses a steady-state distribution.

The long-run behavior of a deterministic BN depends on the initial state and the network will eventually settled down and cycle endlessly through a set of states called an *attractor cycle*. The set of all initial states that reach a particular attractor cycle forms the *basin of attraction* for the cycle. Attractor cycles are disjoint. With perturbation, in the long run the network may randomly escape an attractor cycle, be reinitialized, and then begin its transition process anew.

## 3. QUANTIFYING THE DIFFERENCE BETWEEN NETWORKS

### Distance Functions

To discuss validity, we must first discuss the manner in which we are to compare two networks. Given networks *H* and *M*, we need a function, µ(*M*, *H*), quantifying the difference between them. We require that µ be a *semi-metric*, meaning that it satisfies the following four properties:

1. $\mu \left(M,H\right)\geq 0,$
2. $\mu \left(M,M\right)=0,$
3. $\mu \left(M,H\right)=\mu \left(H,M\right)\left[\mathrm{symmetry}\right],$
4. $\mu \left(M,H\right)\leq \mu \left(M,N\right)+\mu \left(N,H\right)\left[\mathrm{triangle inequality}\right]$.
  As a semi-metric, µ is called a *distance function*. If µ should satisfy a fifth condition,
5. $\mu \left(M,H\right)=0\Rightarrow M=H,$

then it is a *metric*. A distance function is often defined in terms of some characteristic, by which we mean some structure associated with a network, such as its regulatory graph, steady-state distribution, or probability transition matrix. This is why we do not require the fifth condition, $\mu \left(M,H\right)=0\Rightarrow M=H,$ for a network distance function. 

If we want to approximate one network by another, say for reasons of computational complexity, then a distance function can be used to measure the goodness of the approximation. If *M_1_* and *M_2_* are two approximations of network *H*, then *M_1_* is a better approximation than *M_2_* relative to µ if µ $\mu \left(M_{1},H\right)<\mu \left(M_{2},H\right)$.

Because a network distance function need only be a semi-metric, one must be careful in applying propositions from the theory of metric spaces. For instance, in a metric space, if a sequence of points in the space is convergent, then the limit of the sequence is unique. When the points are networks, this is not necessarily true. A sequence of networks can converge to two distinct networks: {*H_i_*} can converge to both *M* and *N*, with M≠N.

### Rule-Based Distance

For Boolean networks (with or without perturbation) possessing the same gene set, a distance is given by the proportion of incorrect rows in the function-defining truth tables. Denoting the state functions for networks *H* and *M* by **f** = (*f*_1_, *f*_2_,…, *f_n_*) and **g** = (*g*_1_, *g*_2_,…, *g_n_*), respectively, since there are *n* truth tables consisting of 2^*n*^ rows each, this distance is given by

(7) $$\mu_{\mathrm{fun}}\left(M,H\right)=\frac{1}{n2^{n}}\sum_{i=1}^{n}\sum_{k=1}^{N}I\left[g_{i}\left(x_{k}\right)\neq f_{i}\left(x_{k}\right)\right]$$

where *I* denotes the indicator function, *I*[*A*] = 1 if *A* is a true statement and *I*[*A*] = 0 otherwise [6]. If we wish to give more weight to those states more likely to be observed in the steady state, then we can weight the inner sums in Eq. 7 by the corresponding terms in the steady-state distribution, **π** = (π_1_, π_2_,…, π_*N*_). For Boolean networks without perturbation, µ_*fun*_ is a metric. If there is perturbation, then µ_*fun*_ is not a metric because two distinct networks may be identical with regard to the rules but possess different perturbation probabilities. 

### Topology-Based Distance

If one’s focus is on the topology of a network, then a straightforward approach is to construct the adjacency matrix. Given an *n*-gene network, for *i*, *j* = 1, 2,…, *n*, the (*i*, *j*) entry in the matrix is 1 if there is a directed edge from the *i*th to the *j*th gene; otherwise, the (*i*, *j*) entry is 0. If **A** = (*a_ij_*) and **B** = (*b_ij_*) are the adjacency matrices for networks *H* and *M*, respectively, where *H* and *M* possess the same gene set, then the *hamming *distance between the networks is defined by

(8) μhamM,H=∑i,j=1naij−bij

Alternatively, the hamming distance may be computed by normalizing the sum, such as by the number of genes or the number of edges in one of the networks, for instance, when one of the networks is considered as representing ground truth. The hamming distance is a coarse measure since it contains no steady-state or dynamic information. Two networks can be very different and yet have $\mu_{\mathrm{ham}}\left(M,H\right)=0$ .

If one of the networks in Eq. 8 is considered as ground truth, then the hamming distance can be reformulated in terms of the numbers of false-negative and false-positive edges. If *H* is the ground-truth network, then a false-negative edge is a directed edge not in *M* that is in *H* and a false-positive edge is directed edge in *M* that is not in *H*. Letting *FN* and *FP* be the numbers of false-negative and false-positive edges, respectively, the hamming distance is given by *FN* + *FP*. Because we are considering directed graphs, an incorrectly oriented edge in *M* between two genes is both a false-negative and false-positive edge, although one can slightly alter the definitions to avoid this kind of double counting. If we were to consider undirected graphs, then this anomaly would not occur because an edge would either be present or absent. In this case, the hamming distance is still defined by Eq. 8 but the adjacency matrix is symmetric. 

Since our interest is measuring the closeness of an inferred network to the network generating the data, we concentrate on distance functions, in particular, the hamming distance, which has been used for this purpose [7, 8]. Non-distance measures related to the hamming distance have been used in the context of regulatory graphs. Again let *H* denote the ground-truth network. A true-positive edge is a directed edge in both *H* and *M*, and a true-negative edge is a directed edge in neither *H* nor *M* (with analogous definitions holding for undirected graphs). Let *TP* and *TN* be the numbers of true-positive and true-negative edges, respectively. The *positive predictive value* is defined by *TP*/(*TP* + *FP*), the *sensitivity* is defined by *TP*/(*TP* + *F*N), and the *specificity* is defined by *TN*(*TN* + *FP*). These kinds of measures have been used in several regulatory-graph inference papers [8-12] and a study using these measures has been performed to evaluate a number of inference procedures [13]. 

### Transition-Probability-Based Distance 

Distances for Markov networks can be defined *via *their probability transition matrices by considering matrix norms. A *norm* is a function $\left\|•\right\|$ on a linear (vector) space, *L*, such that:

1. $\left\|v\right\|\geq 0,$
2. $\left\|v\right\|=0\Rightarrow v=0,$
3. $\left\|\mathrm{av}\right\|=\left|a\right|\cdot \left\|v\right\|\left[\mathrm{homogeneity}\right],$
4. $\left\|v+w\right\|\leq \left\|v\right\|+\left\|w\right\|\left[\mathrm{triangle inequality}\right].$

Given a norm on *L*, a metric is defined on *L* by $\left\|v-w\right\|$ .

For an *n* x *n* matrix and *r* ≥ 1, the *r*-norm is defined by

(9) $${\left\|P\right\|}_{r}={\left(\sum_{i,j=1}^{n}{\left|p_{\mathrm{ij}}\right|}^{r}\right)}^{1/r}$$

The *supremum* norm is defined by

(10) $${\left\|P\right\|}_{\infty }=\text{max}\left\{\left|p_{\mathrm{ij}}\right|:i,j=1,2,...,n\right\}$$

These norms are well-studied in linear algebra. Each yields a metric defined by ${\left\|P-Q\right\|}_{r\cdot }$ If $P=\left(p_{\mathrm{ij}}\right)$ and $Q=\left(q_{\mathrm{ij}}\right)$ are the probability transition matrices for networks *H* and *M*, respectively, then a network distance function is defined by

(11) $$\begin{array}{cc} \mu_{\mathrm{prob}}^{r}\left(M,H\right)= & {\left\|P-Q\right\|}_{r} \end{array}$$

Whereas ${\left\|•\right\|}_{r}$ defines a matrix metric, $\mu_{\mathrm{prob}}^{r}$ is only a network semi-metric because two distinct networks may have the same transition probability matrix.

### Long-Run Distance

Since steady-state behavior is of particular interest, for instance, being associated with phenotypes, a natural choice for a network distance is to measure the difference between steady-state distributions [14]. If **π **= (π_1_, π_2_,…, π_*N*_) is a probability vector, then its *r*-norm is defined by

(12) $${\left\|\pi \right\|}_{r}={\left(\sum_{i=1}^{N}{\left|\pi_{i}\right|}^{r}\right)}^{1/r}$$

for *r* ≥ 1, and its supremum norm is defined by

(13) $${\left\|\pi \right\|}_{\infty }=\text{max}\left\{\left|\pi_{i}\right|:i=1,2,...,N\right\}$$

If **π **= (π_1_, π_2_,…, π_*N*_) and **ω **= (ω_1_, ω_2_,…, ω_*N*_) are the steady-state distributions for networks *H* and *M*, respectively, then a network distance is defined by

(14) $$\mu_{\mathrm{stead}}^{r}\left(M,H\right)={\left\|\pi -\omega \right\|}_{r}$$

Other norms can be used to define the distance function.

Not all networks possess steady-state distributions. The long-run behavior of a deterministic rule-based network, such as a Boolean network, depends on the initial state. A rule-based finite-value network possesses attractor cycles that characterize its long-run behavior and we can consider comparing this long-run behavior. This can be done by considering the proportion of time spent in a state once an attractor cycle has been entered. For any initial state **x**_*k*_, the network eventually enters the attractor cycle, *C_k_*, whose basin contains **x**_*k*_. An arbitrary state **x**_*j*_ either lies in *C_k_* or it does not. Let *m_k_* denote the number of states in *C_k_* and *p_k_* be the probability that the initial state is **x**_*k*_. We define the long-run probability of **x**_*j*_ by

(15) $$\zeta_{j}=\sum_{K=1}^{N}\frac{p_{k}}{m_{k}}I\left[x_{j}\in C_{k}\right]$$

Letting **ζ** = (ζ_1_, ζ_2_, …ζ_N_), we can proceed analogously to the steady-state case by replacing **π** by  **ζ** to define the *r*-norm, and then define the distance function $\mu_{\mathrm{long}}^{r}\left(M,H\right)$ in the usual way.

Suppose all attractor cycles are singletons, so that *m_k_* = 1. Moreover, suppose we do not know the initial-state probabilities and we set *p_k_* = 1/*N*. If **x**_*k*_ is an attractor, let *b_k_* denote the number of states in its basin; if **x**_*k*_ is not an attractor, let *b_k_* = 0. Then Eq. 15 reduces to ζ_*j*_ = *b_j_*/*N*. To this point, **ζ** = (ζ_1_, ζ_2_,…, ζ_*N*_) describes a probability density because its components sum to 1. Now suppose we ignore the basin sizes so that ζ*_j_* = 1/*N* if **x***j* is an attractor and ζ*_j_* = 0 otherwise. If **ζ** = (ζ_1_, ζ_2_,…, ζ*_N_*) and **ξ **= (ξ_1_, ξ_2_,…, ξ_*N*_) correspond to networks *H* and *M*, respectively, then the network distance induced by the 1-norm is given by

(16) $$\mu_{\mathrm{att}}\left(M,H\right)=\sum_{j-1}^{N}\left|\zeta_{j}-\xi_{j}|=\frac{1}{N}|A_{H}{\Delta A}_{M}\right|$$

where *A_H_* and *A_M_* are the attractor sets for *H* and *M*, respectively,

(17) $$A_{H}\Delta A_{M}=\left(A_{H}-A_{M}\right)\cup \left(A_{M}-A_{H}\right)$$

is the symmetric difference of *A_H_* and *A_M_*, and $\left|•\right|$denotes the number of elements in a set. The distance μ_*att*_(*M*, *H*) compares the attractor sets of the two networks. μ_*att*_(*M*,*H*) = 0 if and only the attractor sets are the same. We have derived μ_*att*_(*M*, *H*) from μ_*long*_(*M*, *H*) assuming only singleton attractors, but μ_*att*_(*M*, *H*) can be applied to any rule-based discrete network.

### Trajectory-Based Distance

Continuing with rule-based finite-value networks, rather than simply focusing on the long-run probabilities, one can take a more refined perspective by considering differences in the trajectories. Continue to let *m_k_* denote the number of states in the cycle *C_k_* for initial state **x**_*k*_ and let *t_k_* be the time it takes **x**_*k*_ to reach *C_k_*. The time trajectory of the network is given by **X**(*t*) = (*X*_1_(*t*), *X*_2_(*t*),…, *X_n_*(*t*)). For a given initial state this trajectory is deterministic. For initial state **x**_*k*_, denote the trajectory by

(18) $$x^{\left(k\right)}\left(t\right)=\left(x_{1}^{\left(k\right)}\left(t\right),x_{2}^{\left(k\right)}\left(t\right),...,x_{n}^{\left(k\right)}\left(t\right)\right)$$

Given the initial state is **x**_*k*_, we define the *amplitude cumulative distribution* of gene *X_i_* by

(19) $$F_{i}\left(z\left|k\right.\right)=\frac{1}{m_{k}}\sum_{t=t_{k}}^{t_{k}+m_{k}-1}I\left[x_{i}^{\left(k\right)}\left(t\right)\leq z\right]$$

This increasing function of *z* counts the fraction of time that $x_{i}^{\left(k\right)}\left(t\right)\leq z$ in the cycle *C_k_*.

Given two attractor cycles, *C_k_* and *C_j_*, resulting from initializations **x**_*k*_ and **x**_*j*_, respectively, we define a distance between the cycles relative to gene *X_i_* using the amplitude cumulative distributions, $F_{i}\left(•|k\right)$ and $F_{i}\left(•|j\right)$ , by

(20) $$\delta_{i}\left(C_{k},C_{j}\right)=\left\|F_{i}\left(•\left|k\right.\right)-F_{i}\left(•\left|j\right.\right)\right\|$$

for some function norm $\left\|•\right\|$ . For example, we could use the *L*_1_ norm

(21) $${\left\|F_{i}\left(\left.•\right|k\right)-F_{i}\left(\left.•\right|j\right)\right\|}_{1}=\int_{0}^{\infty }\left|F_{i}\left(z|k\right)-F_{i}\left(z|j\right)\left|\mathrm{dz}\right.\right.$$

The *L*_1_ norm possesses an interesting interpretation if gene *X_i_* has constant amplitude values, *a* and *b*, on cycles *C_k_* and *C_j_*, respectively. In this case, $F_{i}\left(•|k\right)$ and $F_{i}\left(•|j\right)$ are unit step functions with steps at *a* and *b*, respectively. Hence, in this case the *L*_1_ norm reduces to

(22) $$\delta_{i}\left(C_{k},C_{j}\right)=\left|a-b\right|$$

and gives the distance, in amplitude, between the values of gene *X_i_* on the two cycles. For a Boolean network, $\delta_{i}\left(C_{k},C_{j}\right)=0$ if the gene is either ON or OFF on both cycles and $\delta_{i}\left(C_{k},C_{j}\right)=1$ if *X_i_* is ON for one cycle and OFF for the other (assuming *X_i_* is constant on both cycles).

Considering the full set of genes, we define a distance between two attractor cycles, *C_k_* and *C_j_* by

(23) $$\delta \left(C_{k},C_{j}\right)=\frac{1}{n}\sum_{i=1}^{n}\delta_{i}\left(C_{k},C_{j}\right)$$

Now consider two networks, *M* and *H*, having the same genes. We define the distance between M and H as the expected distance between attractor cycles over all possible initial states:

(24) $$\mu_{\mathrm{amp}}\left(M,H\right)=E\left[\delta \left(C_{k}^{M},C_{k}^{H}\right)\right]=\sum_{k=1}^{N}p_{k}\delta \left(C_{k}^{M},C_{k}^{H}\right)$$

where $C_{k}^{M}$ and $C_{k}^{H}$ are the attractor cycles corresponding to initialization by state **x**_*k*_ in networks *M* and *H*, respectively [15]. 

### Equivalence Classes of Networks

The previous examples of network distance functions demonstrate a common scenario: a network semi-metric is defined by a metric on some network characteristic, for instance, its regulatory graph, its transition probability matrix, etc. The metric requirement, $\mu \left(M,H\right)=0\Rightarrow M=H$ fails because distinct networks possess the same characteristic. To formalize the situation, let λ_M_ and λ_H_ denote the characteristic λ corresponding to networks *M* and *H*, respectively. If ν is a metric on a space of characteristics (directed graphs, matrices, probability densities, etc.), then a semi-metric μ_ν_ is induced on the network space according to

(25) $$\mu_{v}\left(M,H\right)=v\left(\lambda_{M},\lambda_{H}\right)$$

This is quite natural if our main interest is with the characteristic, not the specific network itself.

Focus on network characteristics leads to the identification of networks possessing the same characteristic. Given any set, *U*, a relation ~ between elements of *U* is called an *equivalence relation* if it satisfies the following three properties for a,b,c∈U:

1. $a∼a\left[\text{reflexivity}\right],$
2. $a∼b\Rightarrow b∼a\left[\mathrm{symmetry}\right],$
3. $a∼b\text{ and }b∼c\Rightarrow a∼c\left[\mathrm{transitivity}\right].$

If *a* ~ *b*, then *a* and *b* are said to be *equivalent.* An equivalence relation on *U* induces a partition of *U*. The subsets forming the partition are defined according to *a* and *b* lie in the same subset if and only if *a ~ b*. The subsets are called *equivalence classes*. The equivalence class of elements equivalent to *a* is denoted by [*a*]^~^. According to the definitions, ${\left[a\right]}^{∼}={\left[b\right]}^{∼}$ if and only if *a* ~ *b* . 

If ν is a semi-metric on a set *U* and we define *a* ~ *b* if and only if ν(*a*, *b*) = 0, then

(26) $$\mu \left({\left[a\right]}^{∼},{\left[b\right]}^{∼}\right)=\nu \left(a,b\right)$$

defines a metric on the space of equivalence classes because $\mu \left({\left[a\right]}^{∼},{\left[b\right]}^{∼}\right)=0\Leftrightarrow \nu \left(a,b\right)=0\Leftrightarrow a∼b\Leftrightarrow {\left[a\right]}^{∼}={\left[b\right]}^{∼}$.

If we define *M* ~ *H* if λ*_M_* = λ*_H_*, then this is a network equivalence relation. If we focus on equivalence classes of networks rather than the networks themselves, we are in effect identifying equivalent networks. For instance, if we are only interested in steady-state distributions, then it may be advantageous to identify networks possessing the same steady-state distribution. 

## 4. INFERENCE PERFORMANCE

An inference procedure operates on data generated by a network *H* and constructs an inferred network *M* to serve as an estimate of *H*, or it constructs a characteristic to serve as an estimate of the corresponding characteristic of *H*. For instance, the data may be used to infer a distribution that estimates the steady-state distribution of *H*. The data could be dynamical, consisting of time-course observations, or it might be taken from the steady state, as with microarray measurements assumed to come from the steady state of some phenotypic class. In the latter case, it makes sense to consider inference accuracy relative to the steady-state distribution of *H*, rather than *H* itself. For full network inference, the inference procedure is a mathematical operation, a mapping from a space of samples to a space of networks, and it must be evaluated as such. There is a generated data set *S* and the inference procedure is of the form ψ(*S*) = *M*. If a characteristic is being estimated, then ψ(*S*) is a characteristic, for instance, ψ*S* = *F*, a probability distribution.

### Measuring Inference Performance Using Distance Functions

Focusing on full network inference, the goodness of an inference procedure ψ is measured relative to some distance, μ, specifically, µ $\mu \left(M,H\right)=\mu \left(\psi \left(S\right),H\right)$ , which is a function of the sample *S*. In fact, *S* is a realization of a random set process, Σ, governing data generation from *H*. In general, there is no assumption on the nature of  Σ. It might be directly generated by *H* or it might result from directly generated data corrupted by noise of some sort. $\mu \left(\psi \left(\Sigma \right),H\right)$ is a random variable and the performance of ψ is characterized by the distribution of $\mu \left(\psi \left(\Sigma \right),H\right)$ , which depends on the distribution of Σ. The salient statistic regarding the distribution of $\mu \left(\psi \left(\Sigma \right),H\right)$ is its mean, $E_{\Sigma }\left[\mu \left(\psi \left(\Sigma \right),H\right)\right]$ , where the expectation is taken with respect to Σ.

Rather than considering a single network, we can consider a distribution, **H**, of random networks, where, by definition, the occurrences of realizations *H* of **H** are governed by a probability distribution. This is precisely the situation with regard to the classical study of random Boolean networks. Averaging over the class of random networks, our interest focuses on

(27) $$\mu^{*}\left(\text{H},\Sigma ,\psi \right)=E_{H}\left[E_{\Sigma }\left[\mu \left(\psi \left(\Sigma \right),\text{H}\right)\right]\right]$$

It is natural to define the inference procedure ψ_1_ better than the inference procedure ψ_2_ relative to the distance μ, the random network H, and the sampling procedure Σ if

(28) $$\mu^{*}\left(\text{H},\Sigma ,\psi_{1}\right)<\mu^{*}\left(\text{H},\Sigma ,\psi_{2}\right)$$

Whether an inference procedure is “good” is not only relative to the distance function, it is relative to how one views the value of the expected distance. Indeed, it is not really possible to determine an absolute notion of goodness.

In practice, the expectation is estimated by an average,

(29) $$\hat{\mu }\left(\text{H},\Sigma ,\psi \right)=\frac{1}{m}\sum_{j=1}^{m}\mu \left(\psi \left(S_{j}\right),H_{j}\right)$$

where *S*_1_, *S*_2_,..., *S_m_* are sample point sets generated according to Σ from networks *H*_1_, *H*_2_,…, *H_m_* randomly chosen from H. 

The preceding analysis applies virtually unchanged when a characteristic is being estimated. One need only replace *H* and H by λ and Λ, where λ and Λ are a characteristic and a random characteristic, respectively, and replace the network distance μ by the characteristic distance.

We next present three examples using previously introduced distance functions to measure inference performance. Algorithm description will be sketchy in order to avoid long digressions from the issue of distance illustration. We defer to the cited literature for details.

#### Example 1.

The Boolean network model has been in existence for a long time and various inference procedures have been proposed [16-18]. One proposed method for Boolean networks with perturbation is based on the observation of a single dynamic realization of the network [6]. This method will be discussed in some detail in Section 5 in regard to consistent inference; for now, we are only concerned with the distance between the inferred network and the original network generating the data, where the distance function is given by µ_*fun*_(*M*, *H*) in Eq. 7. Fig. (**1**) shows the average (in percentage) of the distance function using 80 data sequences generated from 16 randomly generated Boolean networks with 7 genes, perturbation probability *p* = 0.01, uniform connectivity *k* = 2 or *k* = 3, and data sequence lengths varying from 500 to 40,000. The reduction in performance from connectivity 2 to connectivity 3 is not surprising because the number of truth-table lines jumps dramatically.

#### Example 2.

There have been a number of papers addressing the inference of connectivity graphs using information-theoretic approaches [9, 10, 19]. In a study proposing using the minimum description length (MDL) principle to infer regulatory graphs [8], the hamming distance was used to compare the performance of the newly proposed algorithm with an earlier information-theoretic algorithm, called REVEAL [9]. Fig. (**2**) compares the hamming distances between the inferred networks and the corresponding synthetic networks that generated the data relative to increasing sample size. It does so for the REVEAL algorithm and the MDL algorithm using three different settings for a user-defined parameter. The performance measures are obtained by averaging over 30 randomly generated networks, each containing 20 genes and 30 edges, with the distance function being normalized over 30, the number of edges in the synthetic networks.

#### Example 3.

A probabilistic Boolean network (PBN) is a network defined as a collection of discrete-valued networks such that at any point in time one of the constituent networks is controlling the network dynamics [20]. In a context-sensitive PBN there is a binary random variable determining whether there should be a switch of constituent networks at that time point, the modeling assumption being that there are latent variables outside the model network whose changes induce stochasticity into the PBN [21]. Typically, there is also a probability of permutation. This example considers a Bayesian connectivity-based inference procedure for designing PBNs from steady-state data [22]. A synthetic PBN, *H*, composed of two constituent Boolean networks is used to generate a random sample of size 60 from its steady-state distribution and the inference procedure is used to construct a designed PBN composed of ten constituent PBNs (note that the inference procedure does not have input relating to the number of constituent BNs of the generating network). According to definition, the attractors of a PBN are the attractors of its constituent BNs. *H* has six singleton attractors, two of which, call them **x**_*a*_ and **x**_*b*_, contain 0.99 of the steady-state mass. The designed PBN has more attractors, which is not uncommon, but **x**_*a*_ and **x**_*b*_ appear in all ten constituent networks as singleton attractors and they contain 0.78 of the steady-state mass. Since, for PBNs with low probability of network switching almost all of the steady-state mass lies in its attractors [21], $\mu_{\mathrm{stead}}^{1}\left(\psi \left(S\right),H\right)\leq 0.21$ (or approximately so), the maximum 1-norm being 2.

## 5. CONSISTENCY

The greater the amount of data, the better inference one can expect. The hope is that, for large data sets, the inferred network will be close to the generating network. We define an inference procedure, ψ, to be *consistent* if $\mu^{*}\left(\text{H},\Sigma ,\psi \right)\to 0$ as $\left|\Sigma \right|\to \infty$ . We illustrate consistency using Boolean networks with perturbation. We use the inference procedure referred to in Example 1 that applies to a single observed time series [6] and the distance function µ_*fun*_ of Eq. 7.

Owing to perturbation, the network has a steady-state distribution and all states communicate with each other. Hence, given a long time series we are likely to observe most of the states and their corresponding state-to-state transitions **x**_*k*_ → **x**_(*k*)_, for *k* = 1, 2,…, *N*, where **x**_(*k*)_ denotes the next state following **x**_*k*_ under the network state function. If we ignore perturbation, then using the observed state-to-state transitions we can construct a table of state-to-gene transitions of the form **x**_*k*_ → *x_i_*, for *k* = 1, 2,…, *N* and *i* = 1, 2,…, *n*. These define the functions *f*_1_, *f*_2_,…, *f_n_* accordingly. Because the truth table for function *f_i_* has 2^*n*^ rows of the form *f_i_*(**x**_*k*_), some rows may be empty owing to insufficient observations and these rows can be filled in randomly. As the length of the time series increases, the probability of not observing the state **x**_*k*_ goes to 0. Indeed, for any positive integer *c*, if we let η(**x**_k_) denote the number of times **x**_*k*_ is observed in the time series, then $P\left(\eta \left({\text{x}}_{\text{k}}\right)\geq c\right)\to 1$ as $\left|\Sigma \right|\to \infty$ , where the probability is with respect to the time series Σ. 

With perturbation, the state-to-state transitions do not directly define functions because state **x**_*k*_ may transition to more than one state. However, assuming a perturbation probability less than 0.5, the transitions from **x**_*k*_ will be dominated by the single transition determined by the state function **f** and this dominating choice can be used for inference. Letting η_*j*_(**x**_*k*_) denote the number times we observe the transition ${\text{x}}_{k}\to {\text{x}}_{j}$ if $\text{f}\left({\text{x}}_{k}\right)={\text{x}}_{k}$ is the function-defined transition, then

(30) $$P\left(\eta_{\left(k\right)}\left({\text{x}}_{k}\right)>\text{max}\left\{\eta_{1}\left({\text{x}}_{k}\right),\eta_{2}\left({\text{x}}_{k}\right),...,\eta_{N}\left({\text{x}}_{k}\right)\right\}\right)\to 1\text{ as }\left|\Sigma \right|\to \infty$$

Thus, if $\hat{\text{f}}$ denotes the inferred state function, then Pfˆ=f→1 as Σ→∞.Similar asymptotic statements hold for *f*_1_, *f*_2_,…, *f_n_*. This insures that, for any τ>0,PμfunψΣ,H<τ→1 as Σ→∞ for any Boolean network *H*. Since µ_*fun*_(ψ(Σ), *H*) ≤ 1, this is equivalent to EΣμfunψΣ,H→0 as Σ→∞ Finally, if H is the class of all Boolean networks on *n* genes with perturbation probability *p*, then, since H is a finite set,

(31) $$\mu_{\mathrm{fun}}^{*}\left(\text{H},\Sigma ,\psi \right)=E_{\text{H}}\left[E_{\Sigma }\left[\mu_{\mathrm{fun}}\left(\psi \left(\Sigma \right),\text{H}\right)\right]\right]\to 0\text{ as }\left|\Sigma \right|\to \infty$$

and the inference procedure is consistent relative to µ_*fun*_.

The preceding argument assumes that the perturbation probability is known. A modification of the inference procedure yields an estimator for *p* [6]; however, if *p* is also being estimated, then the model space H is no longer finite and the consistency proof has to be modified. We do not believe this is the proper place to go into such mathematical issues.

## 6. APPROXIMATION

Inference performance is evaluated based on the ability of an inference procedure to identify the network from which the data have been derived. This can only be done exactly if the data-generating network is known. Suppose we do not know the random network, H, generating the data for which we want to evaluate the inference procedure, ψ, but know a network *N* that we believe to be a good approximation to the networks in H. We might then compare the inferred network to *N*. In effect, such a comparison is approximating $\mu^{*}\left(\text{H},\Sigma ,\psi \right)$ by $E_{\Sigma }\left[\mu \left(\psi \left(\Sigma \right),N\right)\right]$ .

The key issue is approximation accuracy. The triangle inequality implies

(32) $$\mu \left(\psi \left(S\right),N\right)-\mu \left(N,H\right)\leq \mu \left(\psi \left(S\right),H\right)\leq \mu \left(\psi \left(S\right),N\right)+\mu \left(N,H\right)$$

for any sample set *S* and H∈H . Hence,

(33) $$\begin{array}{c} E_{\Sigma }\left[\mu \left(\psi \left(\Sigma \right),N\right)\right]-E_{\text{H}}\left[\mu \left(N,\text{H}\right)\right]\\ \leq E_{\text{H}}\left[E_{\Sigma }\left[\mu \left(\psi \left(\Sigma \right),\text{H}\right)\right]\right]\\ \leq E_{\Sigma }\left[\mu \left(\psi \left(\Sigma \right),N\right)\right]+E_{\text{H}}\left[\mu \left(N,\text{H}\right)\right] \end{array}$$

If *E*_H_[µ(*N*, H)] ≈ 0, meaning that *E*_H_[µ(*N*, H)] is small, then the preceding inequality leads to the approximate inequality

(34) $$E_{\Sigma }\left[\mu \left(\psi \left(\Sigma \right),N\right)\right]\leq_{\approx }\mu^{*}\left(\text{H},\Sigma ,\psi \right)\leq_{\approx }E_{\Sigma }\left[\mu \left(\psi \left(\Sigma \right),N\right)\right]$$

Thus, if *E*_H_[µ(*N*, H)] ≈ 0, then

(35) $$\mu^{*}\left(\text{H},\Sigma ,\psi \right)\approx E_{\Sigma }\left[\mu \left(\psi \left(\Sigma \right),N\right)\right]$$

and it is reasonable to judge the performance of ψ relative to H by $E_{\Sigma }\left[\mu \left(\psi \left(\Sigma \right),N\right)\right]$ . On the other hand, if *E*_H_[µ(*N*, H)] is not small, then both bounds in Eq. 33 are loose and nothing can be asserted regarding the performance of ψ relative to the data sets on which it is being applied. Therefore, unless *E*_H_[µ(*N*, H)] is small, the entire validation procedure is flawed because the approximation of H by *N* is confounding the procedure. In addition, if $E_{\text{H}}\left[\mu \left(N,\text{H}\right)\right]\approx 0,$ one still has to estimate $E_{\Sigma }\left[\mu \left(\psi \left(\Sigma \right),N\right)\right],$ which generally means that the number of sample sets is sufficiently large that the expectation is well-estimated by the average distance.

The preceding approximation methodology is common in the literature. A proposed inference procedure is applied to one or more real data sets. The inferred network is compared, not to the unknown random network generating the data, but to a model network that has been human-constructed from the literature (and implicitly assumed to approximate the data-generating network). For instance, a directed graph (adjacency matrix), **A**, is constructed from relations found in the literature and the hamming distance is used in the approximating expectation, $E_{\Sigma }\left[\mu_{\mathrm{ham}}\left(\psi \left(\Sigma \right),\text{A}\right)\right],$ in Eq. 35. The aim is to compare the result of the inference procedure to some characteristic related to existing biological knowledge. The problem is that the constructed regulatory graph may not be a good approximation to the regulatory graph for the system generating the data. This can happen because the literature is incomplete, there are insufficiently validated connections reported in the literature, or the conditions under which connections have been discovered, or not discovered, in certain papers are not compatible with the conditions under which the current data have been derived. As a result of any of these situations, the overall validation procedure is confounded by the precision (or lack thereof) of the approximation.

## 7. VALIDATION FROM EXPERIMENTAL DATA

Another form of approximation results from using experimental data for validation rather than synthetic data generated from a known, ground-truth model. In this situation, there is a *test-data *sampling procedure generating data from which an estimate of the desired characteristic corresponding to the underlying physical network is formed. Validation is then *via *the random variable $\mu \left(\psi \left(\Sigma \right),\xi \left(\Omega \right)\right),$ where Σ is the *training-data* sampling procedure used to design the network and Ω is a real-data *test-sampling* procedure to validate the designed network by direct construction of the characteristic *via *independent sampling. To simplify the notation we consider a single underlying network *H* rather than a random network H. In this situation, $E_{\text{H}}\left[\mu \left(N,\text{H}\right)\right]$ in Eq. 33 is replaced by $\mu \left(\xi \left(\Omega \right),\lambda_{H}\right)$ , where λ*_H_* is the characteristic for *H*, and Eq. 33 takes the form

(36) $$\begin{array}{c} E_{\Omega }\left[E_{\Sigma }\left[\mu \left(\psi \left(\Sigma \right),\xi \left(\Omega \right)\right)\right]\right]-E_{\Omega }\left[\mu \left(\xi \left(\Omega \right),\lambda_{H}\right)\right]\\ \leq E_{\Sigma }\left[\mu \left(\psi \left(\Sigma \right),\lambda_{H}\right)\right]\\ \leq E_{\Omega }\left[E_{\Sigma }\left[\mu \left(\psi \left(\Sigma \right),\xi \left(\Omega \right)\right)\right]\right]+E_{\Omega }\left[\mu \left(\xi \left(\Omega \right),\lambda_{H}\right)\right] \end{array}$$

If *E*_Ω_[µ(ξ(Ω), λ*_H_*)] ≈ 0, then

(37) $$\mu^{*}\left(H,\Sigma ,\psi \right)\approx E_{\Omega }\left[E_{\Sigma }\left[\mu \left(\psi \left(\Sigma \right),\xi \left(\Omega \right)\right)\right]\right]$$

If ξ is a consistent estimator of λ*_H_*, so that $E_{\Omega }\left[\mu \left(\xi \left(\Omega \right),\lambda_{H}\right)\right]\approx 0$ for large test samples, then, on average, the approximation is good. 

	Consider what happens if one only has data to estimate (train) the model, which may happen when data are limited on account of cost or the availability of samples. In this case, one tests on the same data, thereby having Ω = Σ in Eq. 36 and the *resubstitution *estimate, $E_{\Sigma }\left[\mu \left(\psi \left(\Sigma \right),\xi \left(\Sigma \right)\right)\right]$ , in Eq. 37. If ξ is a consistent estimator of λ*_H_* and the single training sample is large, then the conclusion of Eq. 37 again holds. But we do not have a large sample. Hence, Eq. 36 cannot be used to insure good average performance. But it also cannot be used to insure good performance when there is a small independent test-data sample. In the independent case, we are concerned with the absolute difference

(38) $$\Delta_{\mathrm{test}}=\left|E_{\Sigma }\left[\mu \left(\psi \left(\Sigma \right),\lambda_{H}\right)\right]-E_{\Omega }\left[E_{\Sigma }[\mu \left(\psi \left(\Sigma \right),\xi \left(\Omega \right)\right)\right]]\right|$$

When the same data are used for training and testing, our interest is with

(39) $$\Delta_{\mathrm{train}}=\left|E_{\Sigma }\left[\mu \left(\psi \left(\Sigma \right),\lambda_{H}\right)\right]-E_{\Sigma }\left[\mu \left(\psi \left(\Sigma \right),\xi \left(\Sigma \right)\right)\right]\right|$$

As with classification, where resubstitution error estimation is usually biased low owing to overfitting by the classification rule, in the case of network validation, resubstitution is risky because the characteristic of the designed network is being compared to a characteristic inferred from the same data with which the network has been designed. According to Eq. 36, as in the case of classification, this is not a problem for large samples, but it can be a serious problem for small samples because overfitting can cause Δ_*train*_ to be much less than Δ_*test*_. Whereas substantial effort has gone into studying these kinds of problems in pattern recognition, there appears to be an absence of the analogous study for network validation.

### Example 4.

An attractor-preserving inference method for PBNs based on steady-state data has been proposed and applied to PBNs [23]. A PBN has been designed from cDNA microarray data using 7 genes: WNT5A, pirin, S100P, RET1, MART1, HADHB, and STC2. The steady-state distribution of the designed network has been compared to the histogram of the data, the histogram serving as an estimate of the steady-state distribution of the underlying physical network. Fig. (**3**) illustrates the comparison of the portion of the steady-state distribution corresponding to the data states with the data histogram. Referring to Eq. 14, the 1-norm and 2-norm yield the resubstitution error distances $\mu_{\mathrm{stead}}^{1}\left(\psi \left(S\right),\xi \left(S\right)\right)=0.45$ (out of a maximum of 2) and $\mu_{\mathrm{stead}}^{2}\left(\psi \left(S\right),\xi \left(S\right)\right)=0.1262,$ respectively, the latter being the root-mean-square error. 

## 8. CONCLUSION

This paper has proposed a mathematically rigorous framework for the validation of inference procedures for gene regulatory networks and has illustrated this framework employing validation methods used in the literature. Owing to the central role of regulatory networks in systems biology and the need to apply inference procedures to the massive data sets resulting from high-throughput technologies, validation cannot be left to *ad hoc* methods whose own performances are not understood. A formal framework is necessary. As should be clear from the paper, a great deal of work needs to be done to establish the properties of inference procedures under various conditions, such as the sampling procedure, model class, and validation criterion (distance function). Absent rigorous results in this regard, proposed inference procedures will remain speculative and the quality of their performances unknown. A sound epistemology will be lacking.

## Figures and Tables

**Fig. (1):**
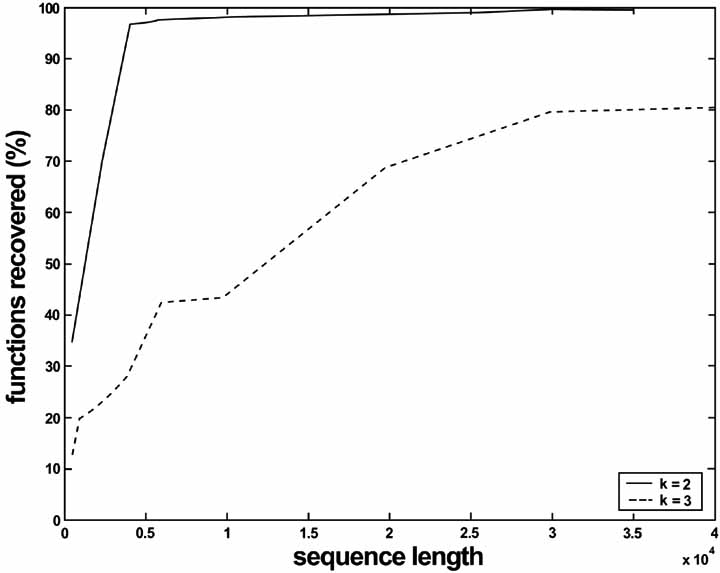
Rule-based distance performance for Boolean-network inference for connectivity *k* = 2, 3.

**Fig. (2):**
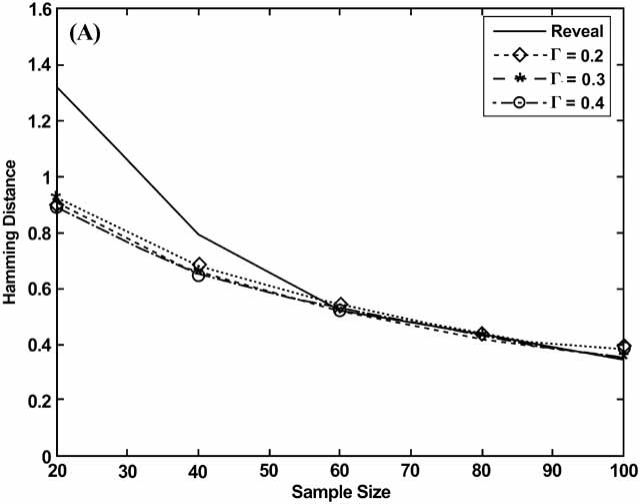
Hamming distance performance for inferring regulatory graphs using information theory.

**Fig. (3):**
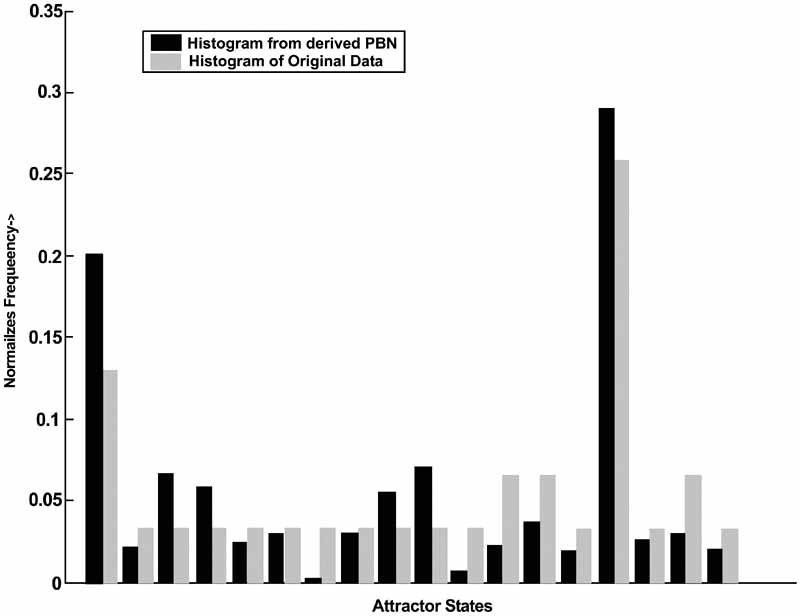
Comparison of steady state distribution for a designed network and data histogram.
